# Diagnosis of Snakebite and the Importance of Immunological Tests in Venom Research

**DOI:** 10.3390/toxins6051667

**Published:** 2014-05-23

**Authors:** R. David G. Theakston, Gavin D. Laing

**Affiliations:** Alistair Reid Venom Research Unit, Liverpool School of Tropical Medicine, University of Liverpool, Liverpool L3 5QA, UK; E-Mail: gavin.laing@liverpool.ac.uk

**Keywords:** snakebite, clinical diagnosis, laboratory diagnosis, biodetection, antivenom, pharmacokinetics, first aid, epidemiology, venom components

## Abstract

In many cases of envenoming following snake bite, the snake responsible for the accident remains unidentified; this frequently results in difficulty deciding which antivenom to administer to the systemically-envenomed victim, especially when only monospecific antivenoms are available. Normally the specific diagnosis of snake bite can be conveniently made using clinical and laboratory methods. Where clinical diagnosis depends upon the recognition of specific signs of envenoming in the patient, laboratory diagnosis is based on the changes which occur in envenomed victims including the detection of abnormalities in blood parameters, presence/absence of myoglobinuria, changes in certain enzyme levels, presence/absence of neurotoxic signs and the detection in the blood of specific venom antigens using immunologically-based techniques, such as enzyme immunoassay. It is the latter which is the main subject of this review, together with the application of techniques currently used to objectively assess the effectiveness of new and existing antivenoms, to assess first aid measures, to investigate the possible use of such methods in epidemiological studies, and to detect individual venom components. With this in mind, we have discussed in some detail how such techniques were developed and how they have helped in the treatment of envenoming particularly and in venom research in general.

## 1. Introduction

“Slash, suck out the venom and apply a tourniquet”—It was partly to challenge this dangerous historical advice that many scientists throughout the world, interested in the treatment of snakebite and other venomous bites and stings, united in a common aim of improving diagnosis and treatment. In snake bite, it is often difficult for clinicians treating patients to determine the species responsible for envenoming, thus making treatment with the correct antivenom more difficult, especially in regions where only monospecific antivenoms are available. This was one of the major reasons which inspired the development of sensitive assay techniques using immunodiagnostic and other laboratory-based methods. Early in investigative studies, it was shown that immunodiagnosis using enzyme immunoassay (EIA) or enzyme-linked immunosorbent assay (ELISA) was useful for the identification of the species responsible for envenoming and also for the detection of specific venom antibody [1]; this followed the detection of venom using radioimmunoassay (RIA) developed by Sutherland’s group in Australia [2,3]. Later, this group also used EIA, which proved to be much cheaper than RIA and obviously did not require the use of radioisotopes [4]. The method enabled the recognition of accurate diagnostic patterns of envenoming by different, sometimes closely related, snake species. Initially, however, a result could only be obtained within a matter of hours rendering an urgent requirement for a more rapid test which would need to provide a reliable diagnostic result within a few minutes of taking a blood sample from the envenomed victim. Only then could the assay system become appropriate for actual early treatment of the patient with antivenom. Such a rapid test has been developed in Australia but, unfortunately, this is considered too expensive and has problems relating to sensitivity [5]. The value of EIA in the study of new and existing antivenoms is that it provides an important objective assessment of antivenom efficacy; as studies mentioned in this review demonstrate, it has proved a useful tool in supplementing clinical observations following antivenom administration after snake bite. Recent advances in the use and development of EIA have added enormously to its use in the field of venom research [6]. The value of EIA in evaluating currently available and novel first aid measures may also prove invaluable both now and in the future, as well as its application in other aspects of venom research.

## 2. Background

**Figure 1:**
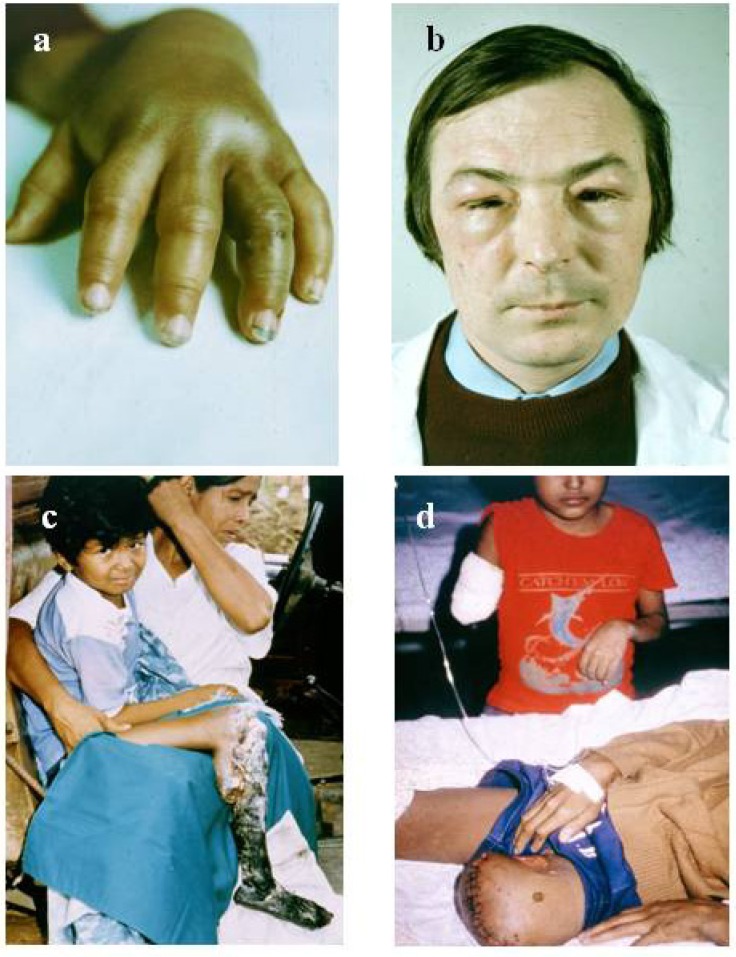
(**a**) Swelling of hand following a viper bite on the third finger of the left hand. Fang marks can be observed (courtesy, Hugh Alistair Reid); (**b**) Swelling and conjunctivitis 4 hours after venom entered the eyes of this individual following a spit by an African spitting cobra (*Naja nigricollis*); (**c**) Local necrosis following a pit viper bite in Peru (courtesy, David Gaus); (**d**) Amputation in two children as a long-term sequel of pit viper (*Bothrops atrox*) bite in Amazonian Brazil (courtesy, David Alan Warrell).

**Figure 2:**
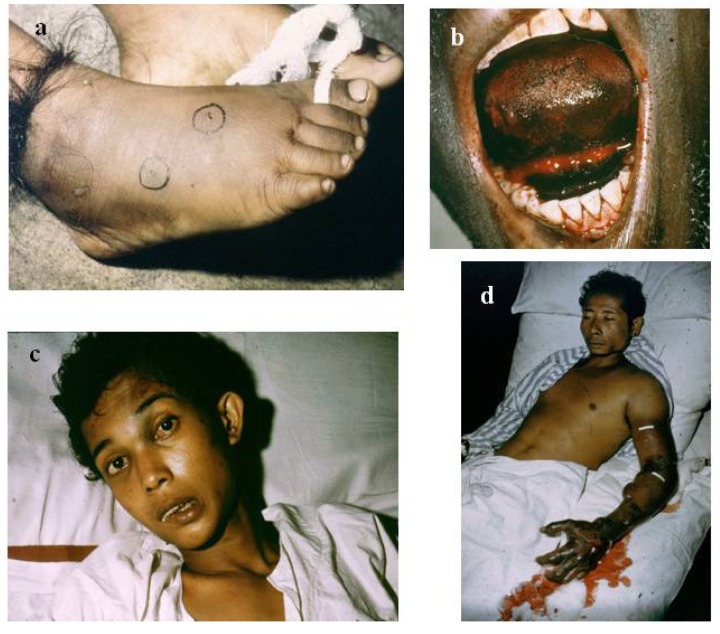
(**a**) Fang marks and hair tourniquet following a bite by a large Russell’s viper in Sri Lanka (courtesy, DA Warrell); (**b**) Bleeding under the tongue and from the gum margins (gingival sulci) caused by viper venom (courtesy, DA Warrell); (**c**) Bleeding into the base of the brain (subarachnoid bleed) following admission to hospital 5 days after a viper bite (courtesy, HA Reid); (**d**) Bleeding into the tissues due to the haemorrhagic effect of a viper venom on the blood vessels resulting in hypovolaemic shock. Blistering in the region of the bite site with underlying local necrosis is also shown (courtesy, HA Reid).

**Figure 3:**
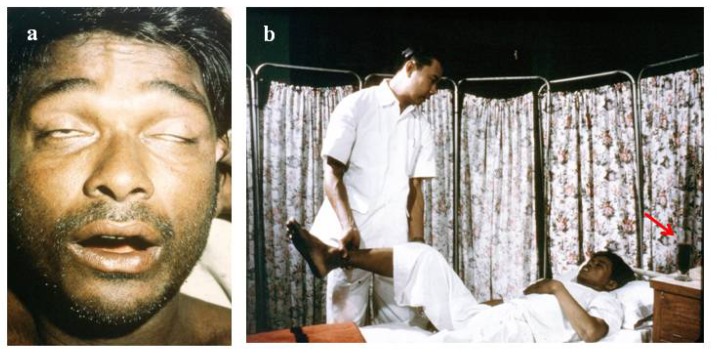
(**a**) Ptosis and inability to extrude the tongue (neurotoxic signs) in a victim of elapid bite (courtesy, DA Warrell); **(b)** Rhabdomyolysis (muscle damage) following a bite by a sea snake. This movement of the leg is extremely painful. Note the presence of myoglobin in the sample of urine by the bedside (arrow) (courtesy HA Reid).

The diagnosis of snake bite or determination of which snake is responsible for envenoming of a victim can be conveniently divided into clinical diagnosis and laboratory diagnosis. Clinical diagnosis depends upon recognising specific signs of envenoming in the patient. This includes local signs such as swelling (Figure 1a,b), blistering (Figure 2d), and local necrosis (Figure 1c,d). More importantly for accurate diagnosis, systemic signs, such as haemorrhage (Figure 2b,c,d), incoagulable blood, and hypovolaemic shock (Figure 2d), are common mainly in viper bite, whereas neurotoxic signs (Figure 3a) occur primarily in elapid bite, and rhabdomyolyis (muscle damage) in sea snake bite (Figure 3b). Indeed, the late Alistair Reid, founder of the Venom Research Unit, Liverpool School of Tropical Medicine, UK, made many of the original observations pertaining to this, although it should be noted that there are exceptions to this rule. For example, some Australian elapid venoms can cause haemorrhage and incoagulable blood in addition to neurotoxicity and the venoms of some vipers, such as the tropical rattlesnake, *Crotalus durissus terrificus*, and the berg adder, *Bitis atropos*, can cause neurotoxic signs in systemically envenomed victims. Local effects, such as necrosis, may occur, especially following viper bites, but this tends to be a slightly later manifestation and is not necessarily diagnostic (Figure 1c,d). Likewise, some cobras are capable of spitting venom; if this enters the eyes it causes a severe local painful conjunctivitis with accompanied swelling (Figure 1b). It should also be noted that the presence or absence of fang marks are not diagnostic although the distance between the fang marks does provide an indication as to the size of the biting snake; however, the detection of fang marks does not necessarily indicate that venom has actually been introduced (Figure 2a). Indeed, in about 50% of bites no venom is injected. More detailed information on clinical diagnosis of snake bite is provided in a recent review [7].

**Figure 4:**
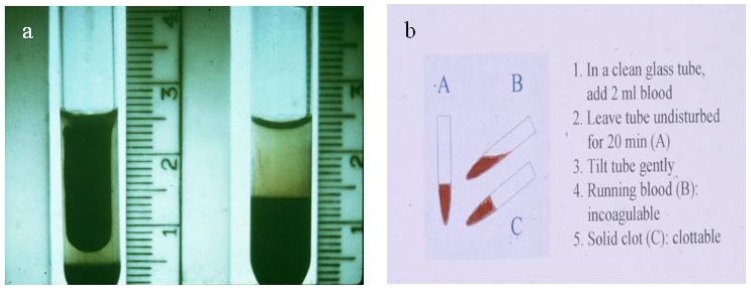
(**a**) Incoagulable blood from a patient with envenoming by a viper. A normal clot is shown on the left; (**b**) The 20 min Whole Blood Clotting Test (WBCT20) for simple bedside detection of venom-induced coagulopathy.

Laboratory diagnosis of snake bite is based on the changes which occur in envenomed victims. These include the detection of abnormal changes in blood parameters (e.g., incoagulable blood (Figure 4a) as examined using the simple bedside 20 min whole blood clotting test, WBCT20 (Figure 4b) [8,9,10], dramatic fall in the platelet count, changes in red and white blood cell counts), presence/absence of myoglobinuria, changes in certain enzyme levels (such as creatine phosphokinase) and the detection in the blood of the victims of specific venom antigens (biodetection methods using immunologically-based techniques). It is the latter point which is one of the main subjects of this review together with the application of other immunological techniques for use in venom research such as the objective assessment of the effectiveness of new and existing antivenoms, the assessment of first aid measures, the possible use of such methods in epidemiological studies and the immunodetection of individual venom components.

## 3. Biodetection Methods Considered for Use in Venom Research

Over the years a number of immunologically-based assay systems have been applied to the detection of specific venom and also to the detection of specific venom antibodies. These include immunodiffusion, immunofluorescence, haemagglutination, immunoelectrophoresis, radioimmunoassay (RIA), enzyme-linked immunosorbent assay (ELISA/EIA), and optical immunoassay, as well as the possible future applications of PCR and antibody microarrays in this respect.

An agar-stabilised precipitation test was first used [11] to detect king cobra (*Ophiophagus hannah*) venom in excised bite site tissue and, later, gel immunodiffusion was used to detect the venoms from four common Nigerian snakes in wound aspirates, blister fluid, sera, and urine samples from envenomed patients [12]. Although generally successful in detecting specific venom, the system was not sensitive enough to detect venom in sera and was, therefore, of limited use.

Immunofluorescence has been used to detect specific venom in tissue samples but not in body fluids [13]. Passive haemagglutination of sheep red cells sensitised to venom by the bis-diazo benzidine coupling procedure was used to demonstrate both venom and antivenom at high dilutions in a test system but problems included instability of the coupling agent and imprecise end-point determination [14]. More recently a single-bead-based immunofluorescence assay has been developed for the detection of venom with a detection sensitivity of 5–10 ng/mL within a 3 h assay time [15]. 

Immunoelectrophoresis was also used but was found to be unlikely to be of practical use in the routine assay of venom and venom antibodies owing to the high levels of common precipitating bands between venoms and antibodies of closely related species [16,17].

Radioimmunoassay [2,18,19] was used to detect venom in the serum of envenomed animals and patients but, although highly sensitive, the method proved to be impractical in patients as well as being very expensive, requiring specialised and elaborate reading equipment for measuring isotope levels in addition to the problems related to the short half-life of ^125^I. It was stressed that its use was primarily as a research tool [3].

**Figure 5:**
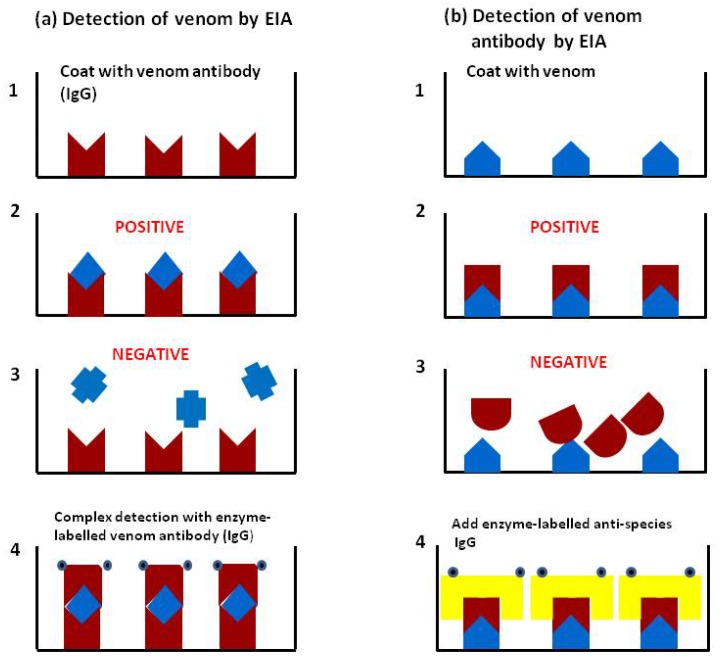
(**a**) Detection of venom by EIA (ELISA): (**1**) Well of microtitre plate is coated with specific venom antibody IgG (or other immunoglobulin fragment such as Fab or F(ab^/^)_2_); (**2**) Patient’s sample containing specific venom is added to the well and binds to the antibody; (**3**) Patient’s sample containing a different venom is added and the venom is not recognised by the antibody and therefore does not bind; (**4**) In the positive sample the antigen/antibody complex is detected using enzyme-labelled antibody IgG; (**b**) Detection of venom antibody by EIA: (**1**) Plate is coated with venom; (**2**) Patient’s sample containing specific venom antibody added and binds to the venom; (**3**) In negative sample no binding occurs; (**4**) In the positive sample, venom/antibody complex is detected using enzyme-labelled anti-species IgG. It should be noted that there is a washing step between each stage of the assay to remove unbound components.

Theakston and colleagues [1] first reported the use of enzyme-linked immunosorbent assay (ELISA) or EIA (enzyme immunoassay), using the double sandwich technique performed in 96 well Microtitre plates [20] for the detection of specific venom and the indirect method for detection of specific antibody (including antivenom) in the blood of envenomed victims. The principle of the technique is based on the linkage of soluble antigens to an insoluble solid phase (the wells of the plate) in such a way that the reactivity of the immunological components is retained. The double sandwich technique consists of binding specific venom antibody to the solid phase followed by a washing step to remove unbound material and subsequent addition of test material containing specific venom antigen. The detection of the venom-antibody complex thus formed is carried out, after further washing, by using specific antibody conjugated to an enzyme (such as horseradish peroxidase or alkaline phosphatase) (Figure 5a). Following a further washing stage, substrate specific for the enzyme is added, the amount of hydrolysis (colour change measured either visually or spectrophotometrically) being proportional to the amount of antigen (venom) present in the test sample. The test can also be used for detection and quantification of venom antigen in other body fluids such as urine and blister and wound aspirates. The indirect method for venom antibody (or antivenom) detection consists of binding antigen (immunologically active venom components) to the solid phase followed by incubation with the test sample. If the sample contains antibody against the specific antigen, the combination can be detected using anti-species immunoglobulin (e.g., horse, sheep, human, *etc.*) conjugated to the enzyme marker (Figure 5b). In this case the amount of hydrolysis is proportional to the amount of antibody present. In order to estimate the amounts of venom or venom antibody in the test sample the results (colour intensity) are compared with a standard curve set up on the same plate as the test samples. The basic principle of the method is shown in Figure 5. The sensitivity of the venom assay is in the region of 1 ng/mL serum, but using a modification of the EIA using a biotin/avidin combination the sensitivity can be even further increased [21]. For ease of sample collection in the field, whole blood samples can be placed on filter paper and dried; the blood is then eluted off with phosphate buffered saline in the laboratory and the EIA carried out on the eluate [22,23]. Pre-coated plates can be stored for later use and incubation times can be further reduced, permitting a total assay time of less than 3 hours although this is still not rapid enough for the clinician to decide on whether or not to treat the patient with antivenom.

Ho and colleagues [24] modified EIA by using a specific blocking system thus decreasing the problem of non-specific false positive reactions. Later, modifications of the EIA were developed [5] which were capable of detecting venom, antivenom, and venom-antivenom complexes; these assays investigated the problems associated with poor sensitivity and the occurrence of false positive results arising from high background absorbance [25].

More recently a specific and sensitive optical immunoassay (OIA) for venom detection was developed; this was similar to EIA but based on the principle of the detection of physical changes in the thickness of a molecular film resulting from specific binding events on an optical silicon chip [26]. The reflection of white light through the thin film results in destructive interference of the light from gold to purple-blue depending on the thickness of the thin film formed or the amount of venom in the test sample. A prototype test kit for the simultaneous identification of the snake species causing envenoming and the semi-quantitative detection of venoms from four medically-important snakes from Vietnam was developed and was found to be capable of detecting venom in blood, plasma, urine, wound and blister aspirates, and tissue homogenates. The sensitivity of the test was claimed to be double that of EIA and the time taken to perform the assay was 33 min.

Suntrarachun and colleagues [27] were the first group to investigate the use of the polymerase chain reaction (PCR) to distinguish the venom of the Thai cobra (*Naja kaouthia*) from the venoms of other Thai species using an experimental mouse model. In this early study, the sequences of nucleotide primers for the cobrotoxin-encoding gene from the Chinese cobra (*Naja atra*) were chosen because, at that time, the sequences of *N.kaouthia* were still unknown. In 2005, mitochondrial DNA (mtDNA) sequences from dried snake venom were used [28] and a DNA barcoding system for the precise identification of venoms was also developed. The group proposed the use of mtDNA for PCR to identify venoms which could overcome some problems encountered with methods such as EIA, although a sizeable venom sample would be required to extract a sufficient quantity of mtDNA; also one would need to decide exactly what to PCR [29]. It may not be a practical system at present because of the very small amounts of venom (nanogram quantities) present in the blood of snake bite victims.

The use of antibody microarrays has also been proposed for detecting specific venoms but, to our knowledge, these have not yet been investigated in this respect. To be able to succeed, two key elements are necessary, namely a unique specific antigen in the venom and a unique antibody (monoclonal antibody) representing the unique protein in the venom of interest. From a proteomics point of view, it is known that there are unique peptide spectra that represent a sequence found only in a certain protein, which could then be only in a specific venom. The key is to select the correct protein and then the right peptide and hope that a monoclonal antibody can be made against it and that it would then bind an epitope in a native protein. This would be possible in principle but has not yet been achieved as far as we are aware [29].

## 4. Major Roles of Enzyme-linked Immunosorbent Assay (ELISA) or Enzyme Immunoassay (EIA) in Venom Research

### 4.1. Accurate Retrospective Diagnosis: Which Snake is Responsible for the Accident?

This is the most important direct role of EIA. As stated earlier it is frequently difficult to determine the species of the snake responsible for the bite. This means that the clinician may have problems in deciding on which antivenom to administer; if he or she cannot decide, then a polyspecific antivenom will be given which is produced by hyperimmunising animals (usually horses but sheep, goats, and even dogs and rabbits have also been used) with a mixture of different venoms obtained from snakes within a particular geographical region. In this situation more immune globulin is required than if a monospecific antivenom (antibody raised using a single venom) is used. This may result in the patient receiving more potentially reactive immunoglobulin causing a higher incidence of early anaphylactic reactions due to the presence of immunoglobulin aggregates (Fab or F(ab^/^)_2_) causing the uncontrolled release of histamine from mast cells. Sometimes the victim may also be at an increased risk of late serum sickness reactions caused by the production of humoral antibodies against the antivenom. 

#### 4.1.1. Validation

In order to be able to depend on the results of venom immunoassay, it is vital first to validate the test. This is carried out by performing the venom assay on a number of serum samples from victims envenomed by different snake species. It is obviously essential that samples are assayed blind, the technician having no prior knowledge of the snake species responsible for the accident. The assay is carried out against the venoms from all species of venomous snakes known to be present in the study region. Once all the assays are complete, the results are compared with the taxonomically-identified snakes responsible for each accident. The study shown in Table 1 was performed in Amazonian Ecuador by our group. Each sample was assayed against the venom of four snake species known to occur in the study area (Shell Pastaza, Southern Ecuador). As the snakes here are closely related species, their venoms contain many common antigens resulting in cross reactivity in the EIA. The snake species responsible for the bite in each case is therefore obtained by selecting the result which yields the highest antibody titre. In 95% of cases, it can be seen that the results obtained by EIA using this criterion agreed with the snake species brought.

**Table 1:** Retrospective diagnosis in Ecuador in 57 cases of venomous snakebite.

Species	Snakes brought	Samples EIA positive	Venom levels (range ng/mL)
*Bothrops atrox*	26	26 (100%)	3-466
*B.bilineatus*	25	24 (96%)	22-421
*Porthidium* *hyoprora*	1	1	350
*B.microphthalmus*	1	No assay developed	5 ^*^
*B.taeniatus*	4	3 (75%)	23-225
*Non venomous*	2	0	-
**Total venomous**	**57**	**54 (95%)**	

Note: ^*^ levels measured in *B.atrox* venom assay.

**Figure 6:**
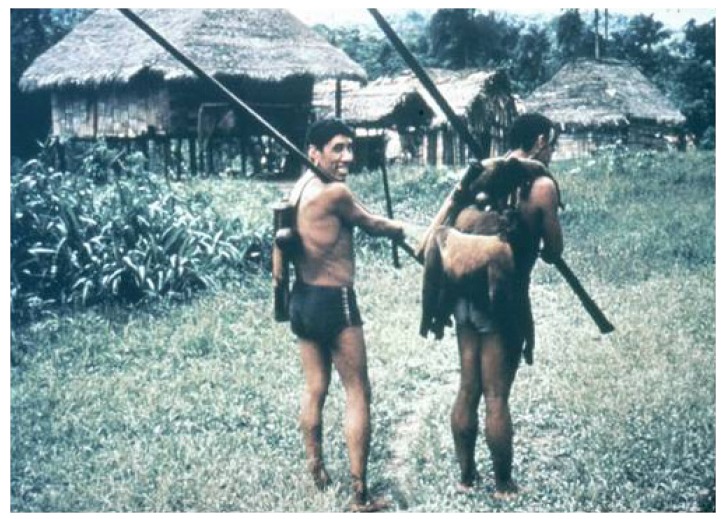
Waorani Indians of eastern Ecuador. About 5% of this tribal group die as a consequence of to snake bite (courtesy James W Larrick).

In another region of Eastern Ecuador, the Waorani tribe suffer a high mortality rate following snake bite due to their mode of living, hunting for food prey in the forest canopy using long blow pipes and poisoned darts (Figure 6). During a survey of health and disease among this population of 612 individuals in 1978 it was estimated that 4.9% of all deaths are due to snake bite, over 45% had experienced at least one bite and almost 95% of the male population had been bitten more than once [30,31].

#### 4.1.2. Application

The identification of the biting species by both lay and many medical personnel is notoriously inaccurate. Individuals involved at the time of the accident almost invariably describe the snake responsible as being “large and black”. EIA enables the reliable and objective determination of the species causing envenoming. 

In North-Central Nigeria (Bambur and Kaltungo, Benue valley) bites by the carpet viper (*Echis ocellatus*) are extremely common but the puff adder (*Bitis arietans*) and the spitting cobra (*Naja nigricollis*) are also implicated in this region. We found that immunodiagnosis in 37 consecutive admissions to one hospital 32 were positive for the venom of *E.ocellatus*, two for that of *B.arietans* and three for the venom of the relatively harmless night adder (*Causus maculatus*) [32]. 

Sri Lanka has one of the highest incidences of snake bite in the world (incidence exceeding 400/100,000 population/year and mortality 6/100,000/year). Our study in this country revealed that out of 94 consecutive hospital admissions in Anuradhapura General Hospital 36 (73% of EIA venom-positive cases) had been envenomed by Russell’s viper (*Daboia russelli pulchella*), five (10%) by the Indian cobra (*N.naja*), five (10%) by the Indian krait (*Bungarus caeruleus*) and three (6%) by the hump-nosed viper (*Hypnale hypnale*). Forty-five patients (48%) showed no signs of either local or systemic envenoming in spite of reporting bites [33]. This supports the observation that about half of snake bite victims receive little or no envenoming.

In Thailand in 1982 we demonstrated that of 82 cases of bites by venomous snakes, 44 were caused by the Thai cobra (*N.kaouthia*) and one by the banded krait (*B.fasciatus*) [34]. The latter patient bitten by *B.fasciatus* died because he had been treated with the wrong monospecific “cobra” antivenom. Further studies in southern Thailand confirmed the diagnosis of envenoming by the Malayan pit viper (*Calloselasma rhodostoma*) in 23 of 46 systemically envenomed patients [7,35]. 

Likewise in Bangladesh, later studies involving EIA accurately identified the species responsible for envenoming in 70 patients with bites by *N.kaouthia* admitted to a large hospital in Chittagong [36,37].

#### 4.1.3. Rapid EIA

For many years there has been a real need for a rapid test for venom detection which is reliable, effective and cheap. Such an assay system (e.g., EIA) would enable the clinician to decide which type of antivenom to use for treating a patient with systemic envenoming by a known snake species. For example, as mentioned earlier and for the reason given above, the most satisfactory antivenom for such a case would be a monospecific antivenom as a smaller volume would be utilised for effective therapy compared with a polyspecific product (providing, of course, that such a monospecific product was available in the hospital dispensary). For example, in Nigeria in 1995 a monospecific *E.ocellatus* antivenom produced by Micropharm Ltd (Newcastle Emlyn, Wales, UK) was available and for the reasons given here was greatly preferable to a polyspecific Pasteur-Merieux Ipser Africa polyspecific (Bitis-Echis-Naja) antivenom [38]. We found that the monospecific antivenom was four-fold more effective in neutralising the venom of Nigerian *E.ocellatus* than the polyspecific product [38].

A Venom Detection Kit (VDK) based on the EIA test [1] was produced by CSL Diagnostics, Melbourne) for use in Australia [4,39] but this was not sensitive enough and was also extremely expensive (£34/test in1995). The test frequently was unable to detect specific venom in serum. A new Snake Venom Detection Kit (SVDK) has been developed more recently which is more sensitive and costs £50/test in 2013 (Williams D, personal communication 2013).

The main problem with the VDK/SVDK is its cost which would be prohibitive in most developing countries where snake bite is a major problem. In Australia it is a useful test but its relative insensitivity, high levels of false positive results [5] and problems relating to cross reactivity [40,41] compared with the standard EIA also leave a lot to be desired although, as stated above, the new assay is more sensitive. It was concluded [42] that the SVDK may assist in regions where the range of medically-important snakes is too broad to permit the use of monospecific antivenoms. A further problem with this kit is that the venom from the bite site swab is the best for obtaining a reliable result; this is presumably because venom concentrations are the highest in this region. However, detection of the venom at the site does not necessarily mean that systemic envenoming has taken place.

### 4.2. The Assessment of New and Existing Antivenoms; How Good is the Antivenom?

This gives us the ability to objectively assess the efficacy of an antivenom by measuring the rate of clearance of venom from the circulation by any antivenom. It also permits the estimation of levels of circulating antivenom at any time after the start of antivenom administration.

#### 4.2.1. Preclinical Assessment of Antivenom Using *in Vivo* and *in Vitro* Tests

Before an antivenom can be used to treat human cases of envenoming, it is essential to first test the product using assays approved by the World Health Organization (WHO) [43,44,45] and also mutually accepted by pharmaceutical companies and Ministries of Health. Such tests must not only examine the efficacy of an antivenom in neutralising the venoms against which it is raised but also, as far as is possible, test the product for safety. The main basic tests include lethality assays and lethality elimination assays carried out in experimental mice. Such tests involve injecting groups of five or six mice with gradually increasing amounts of venom by a predetermined route (usually intravenously) and establishing the amount of venom, which causes the death of half the animals within a group (median lethal dose or LD_50_ of the venom). A multiple of the LD_50_ (usually 2x, 3x, or 5x LD_50_) is then mixed with different amounts of the antivenom under test and the amount of antivenom which results in the survival of half the animals within a group is statistically calculated (median effective dose or ED_50_ of the antivenom). Other *in vivo* and *in vitro* tests commonly used include the elimination of venom-induced pathogenic effects such as those responsible for haemorrhage, coagulopathy (including defibrinogenation), and local necrosis [43,46,47,48,49,50,51,52,53]. *In vivo* tests involve major suffering to the experimental animals involved and currently major efforts are being made to develop non-sentient or other alternative assays [54]. One proposed assay is the use of EIA which, in some circumstances can theoretically replace the standard lethality assay; in one study, good correlation (r = 0.96) was obtained between the rodent ED_50_ test and the ED_50_ as estimated using EIA [55]. It is obviously vital that any alternative assay must provide parallel, statistically comparable results to those obtained using the currently approved rodent-based assays.

#### 4.2.2. Clinical Assessment (Resolution of Clinical Signs in Patients)

This is carried out by clinical observation of the envenomed victim. In systemic envenoming specific antivenom is the only effective therapeutic agent available. If used correctly, it can eliminate the signs of systemic envenoming when given hours or even days after the bite (Figure 2c). It has been shown to eliminate the systemic bleeding caused by the action of venom haemorrhagic metalloproteinases (Figure 2b) within 15–30 min of treatment and to normalise venom-induced coagulopathy (Figure 4a) within two to six hours in some cases of viper bite. Simple bedside tests such as the WBCT20 (Figure 4b) [10] can be used on admission to assess whether or not the blood is coagulable (no antivenom required) or incoagulable due to the action of venom procoagulants or thrombin-like enzymes [56,57]. If the blood is incoagulable, then antivenom should be administered intravenously and the WBCT20 repeated at six hourly intervals until the clotting is normalised. If the blood remains incoagulable after the six hourly WBCT20, then a further dose of antivenom should be given and so on until permanent coagulability is achieved. This test is an excellent means of finding out whether a further dose of antivenom is required. The results obtained using this simple test have been demonstrated to correlate well with low blood fibrinogen levels [10]. Resolution of clinical signs is generally achieved more effectively and at lower dose when a monospecific, as opposed to a polyspecific, antivenom is administered.

Resolution of venom-induced neurotoxicity is simple to record (e.g., elimination of ptosis and respiratory problems). Likewise the cessation of or inhibition of the progression of venom-induced rhabdomyolysis is easily recorded using tests for myoglobin in the urine [58] or estimating CPK-MM levels in the blood [59].

Antivenom is unlikely to be as dramatically effective in eliminating local signs of envenoming such as swelling and/or local necrosis.

#### 4.2.3. Pharmacokinetic Studies (Rate of Venom Elimination from the Circulation)

A large amount of work has been carried out on the pharmacokinetics of envenoming using experimental animals, but relatively little on human victims of envenoming.

In animals, one important study [60] assessed the effect of antivenom in experimentally envenomed rabbits. It was found that following an intramuscular injection of venom, the infusion of a set amount of antivenom caused a redistribution of venom antigens from the extravascular to the vascular space where access to antivenom resulted in neutralisation. It was also found that the difference in the effects of F(ab^/^)_2_ and Fab antivenom fragments could be explained by the different pharmacokinetics of the two fragments; the plasma distribution of venom when Fab antivenom was used was lower than with F(ab^/^)_2_. It was concluded that the *in vivo* neutralisation of venom components by venom antibodies provides an experimental basis for the optimisation of antivenom treatment in humans.

Many other animal-based studies have investigated the pharmacokinetics of envenoming and therapy [61,62,63] but all these used the intravenous route for venom administration which is inappropriate when in human victims venom is almost always either intradermally or intramuscularly.

In our studies EIA has proved to be a very useful method for the objective assessment of antivenom efficacy and dosage [6,35]. It is now possible, as outlined earlier, to detect and quantify specific venom in the blood or other body fluids at any time after the bite (Figure 5a). In addition therapeutic antivenom can be detected and quantified at any time after antivenom administration (Figure 5b). Using this method it is also possible, in addition to detecting whole antivenom IgG, to detect specific antivenom IgG fragments, such as F(ab^/^)_2_ and Fab. 

An assay has recently been developed [64] which demonstrated the presence of venom-antivenom complexes (VAV) in *in vitro* mixtures of venom and antivenom. Measurement of these complexes indicated that in the case of so-called venom recurrence the VAV assay may indicate that there is only VAV present and not free venom. The authors of this study conclude that the VAV assay will provide a useful tool for the investigation of free and bound venom in envenomed patients. This may prove to be the case, but it should be borne in mind that these studies were performed *in vitro* and not *in vivo*. This system was used by the same group in patients envenomed by the Australian brown snake (*Pseudonaja* spp) but at the low venom concentrations that occurred, it was unable to detect VAVs [5].

Rojas and colleagues [65] studied the pharmacokinetics of antivenoms in different animal models. They concluded that equine immunoglobulins have different physico-chemical characteristics which give them the ability to access some body compartments where they can bind toxins. The antivenom concentration in these body compartments also depends on the species of animal used for antivenom production. As equine immunoglobulins are recognised in the homologous model (*i.e.*, horses), or as foreign in heterologous models (e.g., cows, rabbits), immune mechanisms which recognise foreign protein are likely to accelerate the removal of heterologous antivenoms and, thus, affect the way in which the plasma concentrations of antivenom decreases over time. This will distort the pharmacokinetic predictions based on non-compartmental models. 

**Figure 7:**
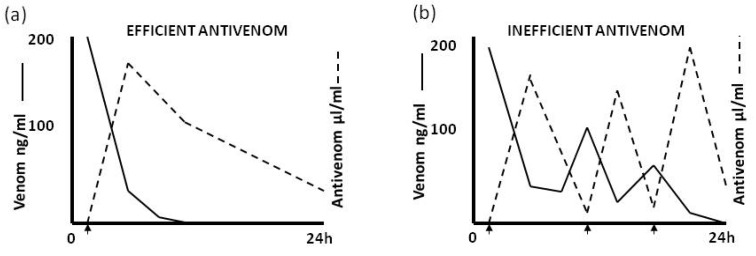
(**a**) The effect of an efficient antivenom and (**b**) an inefficient antivenom on clearance of circulating venom in systemically envenomed patients. Arrows indicate administration of antivenom.

Figure 7 shows the effect of an efficient and inefficient antivenom in eliminating circulating venom. A variety of reasons may be responsible for the poor neutralising activity of an inefficient antivenom. These include a short elimination half-time which renders the antivenom incapable of adequate neutralisation of venom absorbed from the bite site (depot) subsequent to the initial venom antigenaemia or to the fact that the antivenom (e.g., imported Indian Haffkine antivenom used in Sri Lanka and in some African countries) is being produced using venom from snakes of geographical origin different from the area of use [33] and therefore has less neutralising potency. 

**Figure 8:**
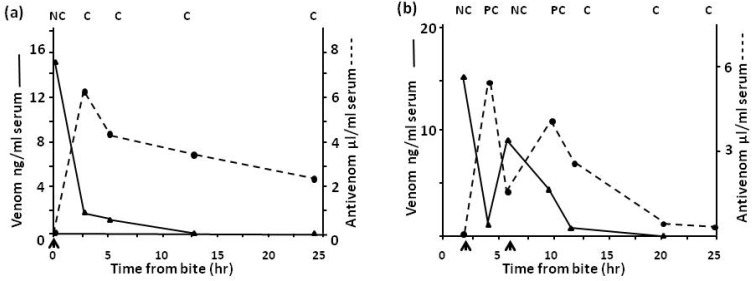
(**a**) Venom levels in a patient treated with one dose of SAIMR monospecific Echis antivenom and (**b**) in a patient treated with Behringwerke polyspecific antivenom. Arrow indicates antivenom given; C = clot, PC = partial clot, NC = no clot.

In Northern Nigeria the saw-scaled or carpet viper, *E.ocellatus*, is by far the commonest cause of snake bite with over 90% of bites in some areas being caused by this species. Figure 8 shows the clearance of venom from the circulation of a patient when an effective antivenom (Figure 8a) and a relatively ineffective antivenom (Figure 8b) is used. Results of immunoassay correlate with the elimination of venom-induced coagulopathy.

**Figure 9:**
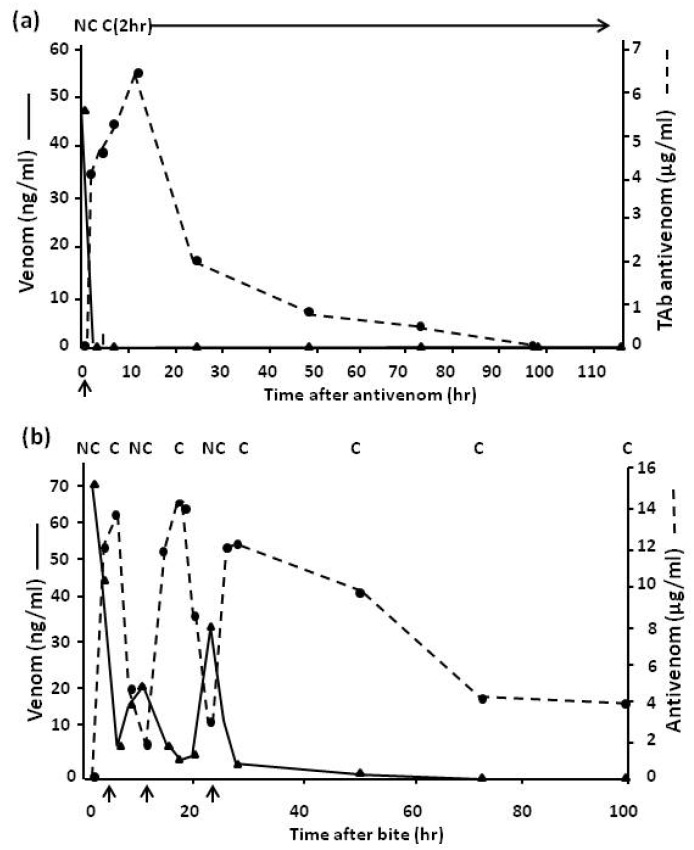
(**a**) Venom levels in a patient treated with one dose of monospecific Sri Lankan Russell’s viper antivenom and (**b**) in a patient treated with Indian Haffkine polyspecific antivenom. Arrow indicates antivenom given; C = clot, NC = no clot.

In Sri Lanka bites by Russell’s viper, *Daboia russelli pulchella*, are common. Figure 9 shows the rapid clearance of venom from the circulation when a more effective monospecific Sri Lankan *D.russelli pulchella* (Therapeutic Antibodies) antivenom (Figure 9a), as opposed to an imported Indian polyspecific (Haffkine) antivenom (Figure 9b), is used.

**Figure 10:**
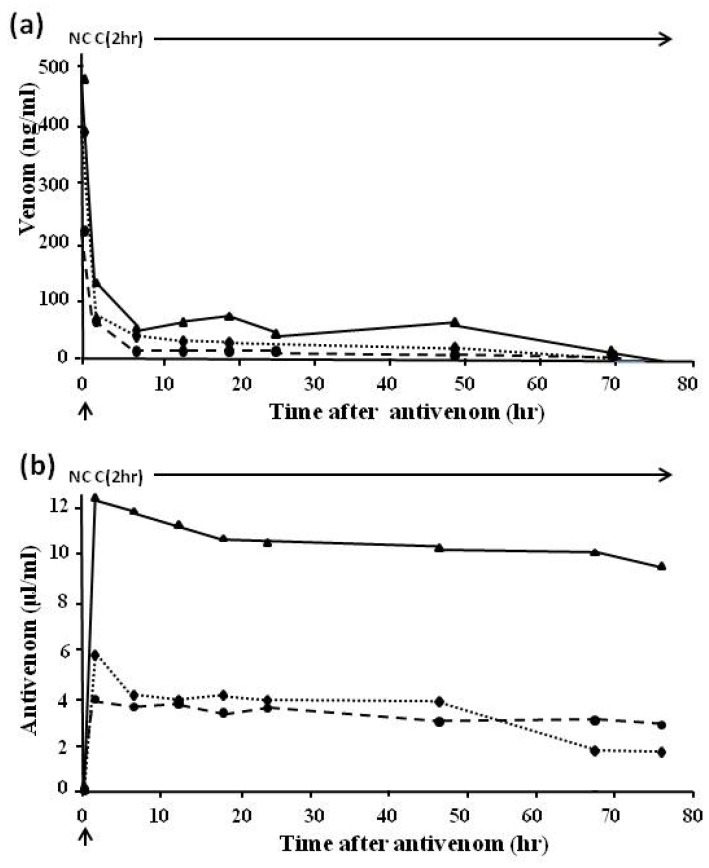
(**a**) Venom and (**b**) antivenom levels in patients bitten by *B.jararaca* and treated with a single dose of eight vials (80 mL) of three Brazilian polyspecific Bothrops antivenoms. Arrow indicates antivenom given; C = clot, NC = no clot; unbroken line, Instituto Butantan antivenom; dotted line, Instituto Vital Brazil antivenom; broken line, Fundação Ezequiel Dias antivenom.

*Bothrops jararaca* is the commonest cause of snake bite in many regions of Brazil. Figure 10 shows venom (Figure 10a) and antivenom (Figure 10b) levels in 32 severely envenomed patients treated with the standard (Brazilian Health Service-approved) dose of eight vials (80 mL) of three different Brazilian antivenoms [66,67]. The results indicated that high levels of antivenom persisted for many hours after venom antigenaemia had been eliminated (as shown both clinically and by EIA). This therefore indicates (1) that the patients appear to be receiving more antivenom that was necessary to neutralise the circulating venom; (2) that it should be possible to reduce the dose to 20 mL or even to 10 mL; and (3) in later studies this proved to be the case [68]. The obvious advantages in reducing the dose of antivenom are a decrease in the incidence of both early, life-threatening, anaphylactic reactions and in delayed serum sickness reactions. This is accompanied by a decrease in the cost of therapy, especially important in a developing country.

Further studies carried out in Papua New Guinea [69], Thailand [7,70], Ecuador [57], and other parts of the world have indicated similar results.

**Figure 11:**
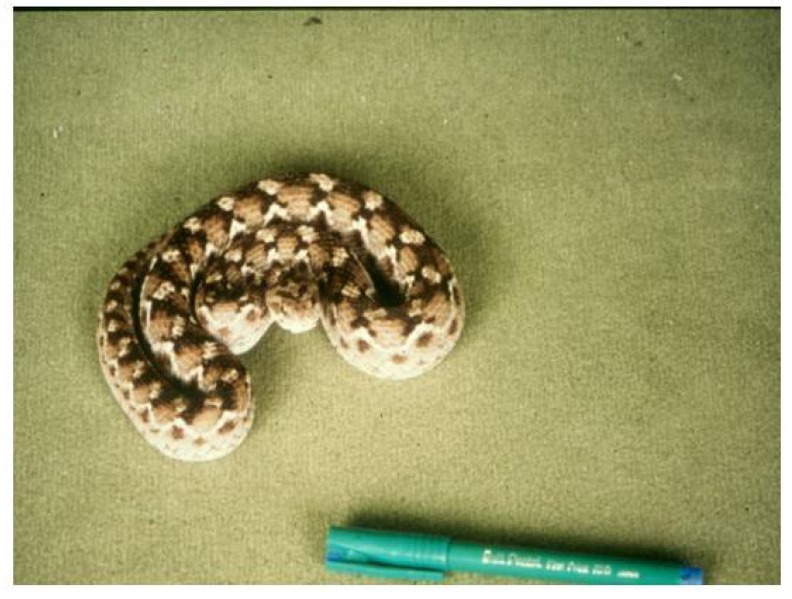
Large specimen of *Echis pyramidum* from Tunisia responsible for a case of severe envenoming which was unresponsive to three different antivenoms with activity against other Echis spp.

**Figure 12:**
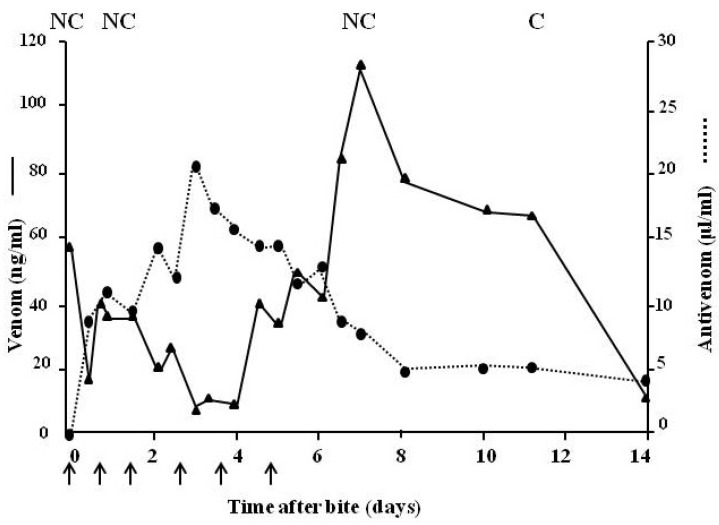
Venom (unbroken line) and antivenom (dotted line) levels in a patient bitten by *E.pyramidum* from Tunisia and treated with six doses (310 mL total) of three different antivenoms with known activity against Echis spp. Arrow indicates antivenom given; C = clot, NC = no clot.

Figure 11 shows a large specimen of *Echis pyramidum* from Tunisia which caused severe systemic envenoming in a bite victim. Figure 12 indicates the sequence of events when the patient was treated with ineffective antivenom. Despite being given a total of 310 mL (six doses) of three different antivenoms (Behringwerke North and West Africa, Pasteur Bitis-Echis-Naja and monospecific South African SAIMR Echis antivenom) together with large amounts of fresh frozen plasma and concentrated clotting factors, venom antigenaemia and coagulopathy persisted for 12–13 days and the patient developed a haemolytic anaemia and mild renal dysfunction. Transient bilateral ptosis was also attributed to envenoming. This has never been reported before in cases systemically envenomed by members of the genus Echis. The venom of the snake responsible for the bite was immunologically distinct from that of Nigerian *E. ocellatus*, and apparently from other *E.pyramidum* “species” and it was clearly not neutralised by any of the three antivenoms which had been administered. Fortunately the patient recovered spontaneously two weeks after the bite [71]. 

### 4.3. Assessment of First Aid Measures

EIA enables the testing of both accepted and traditional methods of first aid therapy. Many of these methods, used both in the past and currently, are of potential danger to the patient.

#### 4.3.1. Possible early First Aid Treatment with Antivenom in the Field

**Figure 13:**
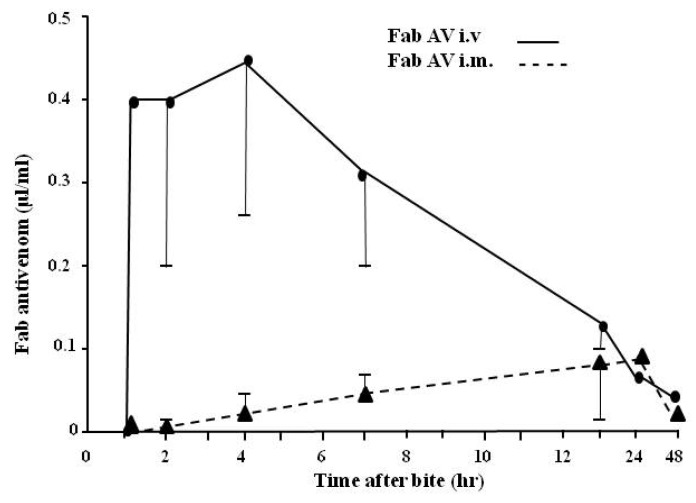
Comparison of the absorption of one ampoule (10 mL) of ovine antivenom given by the iv route (unbroken line) with that given im (broken line) into the thigh muscle. Nine patients were used for each route of injection. Results are mean ± SEM.

A question has often been raised about whether antivenom administered by the easier intramuscular (im), as opposed to the intravenous (iv) route, is effective. If so, the im route could theoretically be used at the pre-hospital stage thus permitting earlier treatment of the patient with antivenom. Figure 13 demonstrates that antivenom given by the im route does not permit high enough levels to be attained for therapeutic reversal of systemic symptoms of envenoming either in relation to time of entry into the circulation or to the levels achieved. The methodology used to carry out this hitherto unpublished study has been reported earlier only using anti-sheep Fab conjugate (see Section 3). 

These results are supported in experimental pharmacokinetic studies carried out on scorpion envenoming in rabbits both before and after antivenom therapy [72]. Following envenoming by the subcutaneous route, intravenous antivenom injection of an appropriate antivenom dose resulted in rapid, complete, and durable neutralization of toxins whereas an injection of the same dose of antivenom intramuscularly resulted in delayed venom neutralization. It was concluded that the antivenom must be injected intravenously to enable efficient immunotherapy. 

#### 4.3.2. The Use of Tourniquets

**Figure 14:**
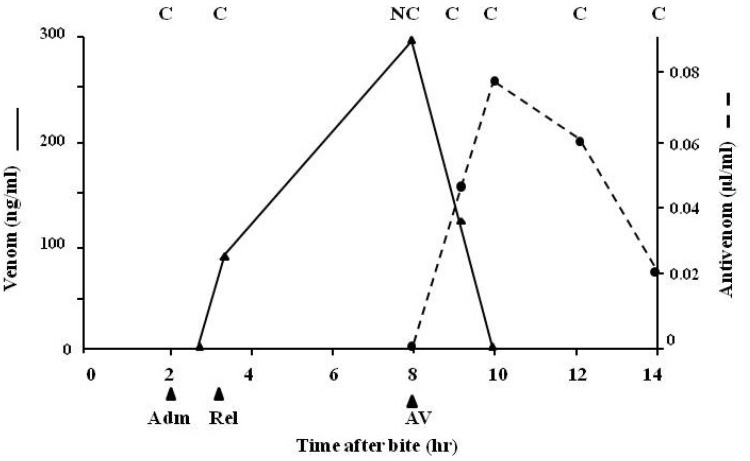
Venom levels (unbroken line) and antivenom levels (broken line) in a patient whose tourniquet was released on admission to hospital. Adm = time of admission to hospital; Rel = release of tourniquet; AV = point at which antivenom was given; C = clot; NC = no clot.

The use of tourniquets (Figure 2a) as a first aid measure in snake bite is highly controversial; tourniquets are frequently not applied correctly and, in bites involving a necrotising venom, they may result in the localisation of high venom concentrations at the bite site causing more severe local necrosis than would have otherwise occurred. Figure 14 indicates rapid release of venom into the circulation following tourniquet removal and the subsequent necessity for immediate antivenom therapy due to the development of clinical symptoms of envenoming.

#### 4.3.3. The Use of Local Remedies in Treatment of Snake Bite

**Figure 15:**
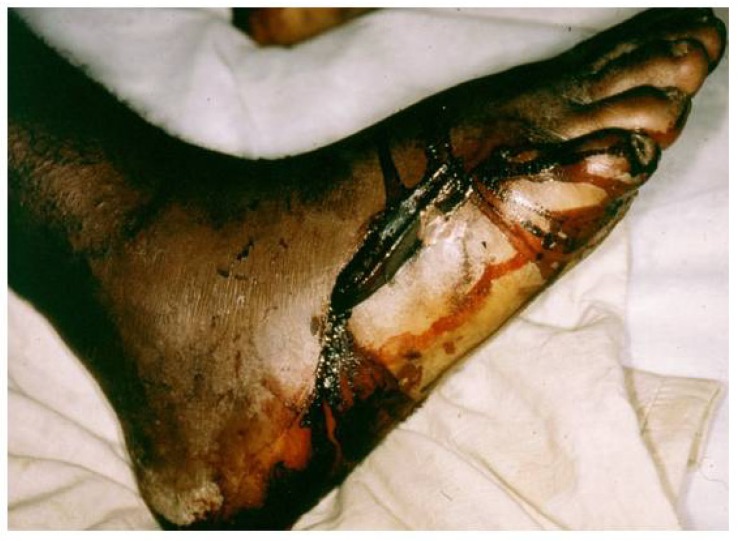
Snake stone applied to the site of the bite on the side of the right foot following a local incision at the site (courtesy, DA Warrell).

The use of local remedies in snake bite is also controversial with most traditional therapies being unlikely to be effective. One of these is the application of the “black snake stone” to the site of the bite, the aim being to extract the venom injected by the snake (Figure 15) via an absorbent medium. When the stone falls off, it is reckoned that the venom has been completely removed from the bite site. It is thought that such objects may be charcoaled bone but it is certain that other applications are also used. Before application of the stone, a cut is made at the bite site thus permitting the stone to adhere. In practice, in a patient with venom-induced coagulopathy and unstable blood vessels due to the action of venom procoagulants and haemorrhagins, this usually results in uncontrolled bleeding at the bite site.

We used an experimental mouse model where a measured minimum lethal dose of venom was injected intradermally and the snake stone was then applied to the bite site. Table 2 indicates that only a minute amount of venom was taken up by the stone when the absorbed venom was determined by EIA. This amount was definitely not enough to prevent the death of the animals, thus, indicating the lack of effectiveness of such a procedure. A more recent study also reported similar findings [73].

**Table 2:** Venom levels in serum, at injection (bite) site and in snakestone 24 h after a sub-lethal dose of venom in experimental mice.

Sample	Venom antigen concentration(mean ± SD (n = 5))
Serum	11.6 ± 5.6 ng/mL
Bite site	21.6 ± 6.7 ng/mL
Stone	0.3 ± 0.4 ng/mL

Other devices such as suction applied to the site are also ineffective and, like the snake stone, may indeed cause adverse effects. In the case of suction devices, which are readily available on the market, there is a high risk of increasing the extent of the local venom-induced necrosis.

Other potential first aid measures could also be readily investigated using EIA. Indeed, Australian workers investigated the efficacy of the pressure immobilisation technique using RIA in experimental monkeys injected with tiger snake (*Notechis scutatus*) venom many years ago [74]. They found that when the injected limb was immobilised and a pressure of 55 mg Hg applied to the injection site, only very low levels of circulating venom were detected. Thus, venom movement into the general circulation can be effectively delayed for long periods by the application of a firm crepe bandage combined with an immobilising splint. The application of pressure alone did not delay venom movement. The same study could just as easily been carried out using EIA. Other methods such as the application of suction devices (considered to be clinically contraindicated as they are said to increase the problems of local venom-induced necrosis) and other devices designed to remove or flush out injected venom could also be readily easily assessed using EIA. 

### 4.4. Epidemiological Studies

**Figure 16:**
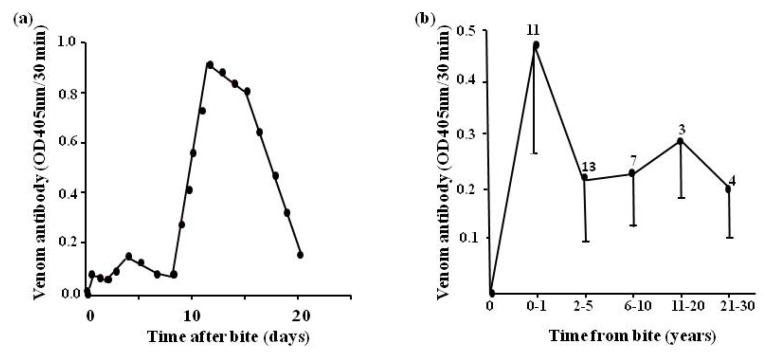
(**a**) humoral venom antibody levels in a patient (LS) envenomed by *E.ocellatus*; (**b**) humoral antibody levels in patients at different times after the bite by vipers in Nigeria. Number of patients at each time range is given above each point (bars represent 2SEM).

We found that using the EIA for detection of specific venom antibody, it is possible to examine the development of humoral venom antibodies [22] in previously envenomed individuals (Figure 16a). Levels are low until about eight days after the bite, rising rapidly to a peak at about 12 days and then falling rapidly. It is possible to also follow the levels of venom antibodies over time from the bite involving envenoming (Figure 16b) and to determine the snake responsible for envenoming in a previous bite [75]. Such a technique has application in epidemiological surveys although it must be stressed that a large control group (minimum 100) of subjects, never previously exposed to snake bite and from the same socio-economic group, must be used as controls. This is because, especially in developing countries where snakebite is a major problem, there may be interference in the assay due to the presence of non-specific antibodies (e.g., rheumatoid factor). The importance of adequate negative controls in such a system cannot be overstressed. For example, one study carried out by our group on the Waorani Indians of Eastern Ecuador (Figure 6) had limited value because controls from Caucasian subjects, as opposed to locals, were used to establish the baseline for the EIAc [76]. 

Current epidemiological studies on snake bite leave a great deal to be desired. Health statistics grossly underestimate its incidence and importance in the rural tropics. Many studies are biased, relying on extrapolation of results from a single area of high snake bite incidence and mortality to a large geographical region [75] as mentioned below; others include only hospital data omitting any reference to snakebite victims who fail to reach hospital. However, if carried out properly, with an adequate number of appropriately selected controls (see above), EIA can contribute to the outcome of more accurate and meaningful epidemiological surveys.

Studies carried out in 1979 in Nigeria using EIA in combination with detailed rural survey procedures [75] indicated that in northern, more densely populated savanna regions (estimated snake bite incidence 48/100,000/year; mortality 5.1%), the main species responsible was the spitting cobra (*N.nigricollis*) whereas in the generally less populated Benue Valley further south the annual snake bite incidence was estimated to be 497/100,000/year with a 12.2% mortality due mainly to the carpet viper (*E.ocellatus*). During this study 531 samples from previous bite victims were assayed for specific venom antibody and 210 (40%) of these were positive (65% against the venom of *E.ocellatus*, 13% against that of *Bitis arietans*, 11% against that of *Causus maculatus*, 8% against that of *N.nigricollis* venom, 3% against *N.haje* venom, and 0% against *B.gabonica* venom). These studies confirmed the importance of *E.ocellatus* reported by Warrell and Arnett in 1976 [77] and also demonstrated the contribution made by other venomous species particularly in milder cases of envenoming and even in severe cases in which victims with long distances to travel were not able to attend hospital or dispensary. On the basis of these figures from rural data it was estimated that snake bite resulted in about 10,000 deaths/year in Northern Nigeria and, if extrapolated to include the entire West African savanna, this implied a mortality of about 23,000 deaths/year caused mainly by *E.ocellatus*. Warrell [78] rightly criticised these figures as being a major overestimation on the ground that unrepresentative areas to permit justifiable extrapolation were selected for the study.

In a further study in French Guiana [79] the incidence of snake bite by defined species proved to be highest in the inhabitants of bush regions and lowest, as expected, in urban areas. Of 43 sera tested for specific venom antibody, 22 (51%) were positive for antibody against local snake venoms.

Other studies combining mouse lethality tests with EIA have also demonstrated that humoral antibodies present in the serum of previous bite victims afford some degree of protection against subsequent exposure to venom [80].

### 4.5. Detection of Individual Venom Components

**Figure 17:**
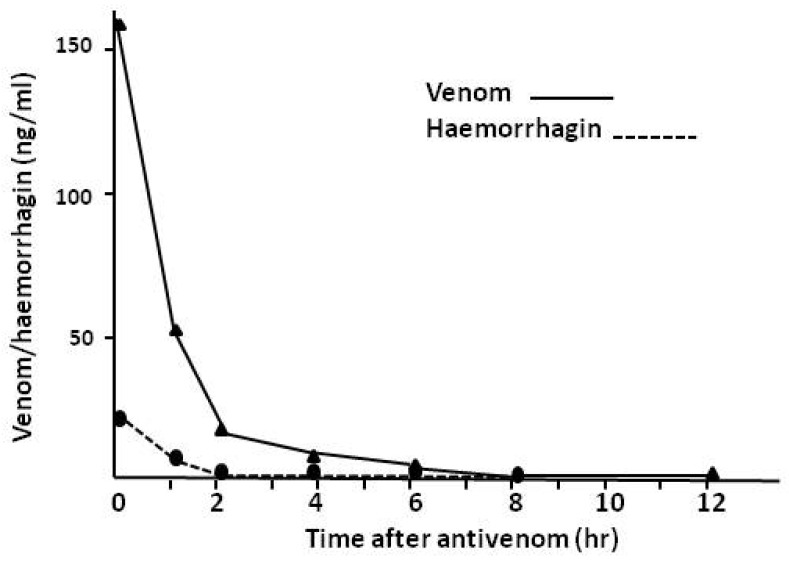
Venom levels (unbroken line) and jararhagin (haemorrhagin) levels (broken line) in serum from a patient.

As venoms contain a multiplicity of both toxic and non-toxic components, EIA permits the detection and quantification of the most important toxic components of a venom and their roles in the pathology and pharmacokinetics of envenoming. Figure 17 shows the detection of jararhagin [81], a haemorrhagic zinc metalloproteinase, in the venom of the Brazilian crotalid, *B.jararaca*. As expected, there is a precise relationship between the extent of systemic bleeding and the presence of jararhagin in the circulation of envenomed victims. For example, in 18 patients with systemic bleeding, venom levels were 357 ± 144 ng/mL serum (±2SEM) with haemorrhagin levels of 45 ± 16 ng/mL serum whereas in 15 patients with no systemic bleeding venom levels were 327 ng/mL ± 196 ng/mL and haemorrhagin levels were 16 ± 8 ng/mL. The differences in haemorrhagin levels were significantly different as would be expected.

## 5. Conclusions

In many cases of envenoming the snake responsible for the accident is not identified, a fact pointed out by Alistair Reid during the course of his research in Malaysia and West Africa, and this was one of his many reasons for encouraging the development of immunodiagnosis in Liverpool. Enzyme immunoassay was developed by his group at the Liverpool School of Tropical Medicine [1] and has proved to be of major use in the biodetection of venom and venom antibodies and in venom research generally. The main application of the EIA is in retrospective identification of the biting species. Although the assay can be shortened to produce a result within three hours, this is still not normally rapid enough to enable the clinician to act on its results. However, together with clinical observations, it enables patterns of envenoming to be established within and between the venomous species present in defined geographical areas. 

A rapid, sensitive, simple and affordable test is still required to enable the clinician to treat the patient with the correct monospecific (or polyspecific) antivenom at the bedside as soon as possible after admission to hospital. Such a kit has been developed in Australia but this is far too expensive for use in most developing countries where snake bite is a major problem of social, medical and economic importance [39]. The kit also has problems relating to decreased sensitivity.

Other potentially useful and tested applications of enzyme immunoassay are described above. These include the ability to examine the pharmacokinetics of envenoming and therapy enabling the objective assessment of both new and existing antivenoms by looking at the rate of elimination of venom from the circulation in systemically envenomed patients.

The method is appropriate for studying the efficacy, or lack of efficacy, of currently available and new first aid approaches. A great deal of damage occurs following the use of many such methods and, importantly, a visit to a traditional healer who applies such techniques drastically delays the arrival of the patient at hospital where approved conventional treatment is available. On the other hand, there should also be awareness that perhaps a traditional pre-hospital treatment method may eventually be discovered which is appropriate and which is assessable using an immunoassay, although this seems unlikely at the present time.

The use of enzyme immunoassay in epidemiological studies of snake bite is potentially extremely useful although the requirement for a powerful control (non-bitten) group of the same socio-economic background is stressed. There is a real need to determine the problem associated with snake bite and other bites and stings throughout the world. Funding bodies require reliable epidemiological information before they will consider supporting a project and in the field of venomous bites and stings this is sorely lacking.

Other methods have been used and suggested to attempt to develop improved methods for studying the pharmacokinetics of envenoming and therapy in envenomed humans; these have been referred to earlier in this review. To date EIA has proved to be the method of preference for the reasons given previously (Section 2).

It is now 50 years since Alistair Reid established the unit in Liverpool and in his honour it was renamed the Alistair Reid Venom Research Unit after his death in 1983. His major contributions to advances in the clinical treatment of envenoming by medically-important snakes and other animals [82], his astute observations on the mode of actions of a wide range of venoms including his role in the development of Arvin from the venom of the Malayan pit viper (*Calloselasma rhodostoma*), continues within the unit named after him.
